# Coin-shaped epithelial lesions following an acute attack of erythema multiforme minor with confocal microscopy findings

**DOI:** 10.4103/0301-4738.58475

**Published:** 2010

**Authors:** Kalpana Babu, Vinay R Murthy, Veeresh P Akki, Venkatesh C Prabhakaran, K R Murthy

**Affiliations:** Vittala International Institute of Ophthalmology & Prabha Eye Clinic and Research Center, Bangalore, India

**Keywords:** Coin-shaped epithelial lesions, confocal microscopy, erythema multiforme minor

## Abstract

We report an interesting ocular finding of bilateral multiple coin-shaped epithelial lesions along with the confocal microscopy findings in a patient following an acute attack of erythema multiforme (EM) minor. A 30-year-old male presented with a history of watering and irritation in both eyes of three days duration. He was diagnosed to have EM minor and was on oral acyclovir. Slit-lamp examination revealed multiple coin-shaped epithelial lesions. Confocal microscopy showed a corresponding conglomerate of hyper-reflective epithelial lesions. The corneal lesions resolved over six weeks with oral steroids and acyclovir. An immunological mechanism is suspected.

Ocular manifestations following Steven-Johnson's syndrome (SJS) is common and have been well-described in literature.[1,2] However, ocular manifestations following an attack of erythema multiforme (EM) minor is rare.[1] We report an interesting ocular finding of multiple coin-shaped epithelial lesions along with the confocal microscopy findings following an acute attack of EM minor.

## Case Report

A 30-year-old male presented to us with a history of watering and irritation in both eyes of three days duration. He was diagnosed to have EM minor following an attack of viral fever with skin eruptions on the face, arms and legs along with shallow, aphthous ulcers, one week back. There was no history of any drug intake leading to the fever and occurrence of skin eruptions. The erythrocyte sedimentation rate (ESR) was 42 mm/h and the total white blood count was 12,000 cells/mm^3^ with predominant neutrophils (72%). Routine urine examination was normal. Blood and urine cultures were negative for bacteria and fungi. Chest X-ray was normal. Antinuclear antibody and rheumatoid factor were negative. He was on oral acyclovir 800 mg five times/day, started by his treating dermatologist, given for a total of seven days.

**Figure 1:**
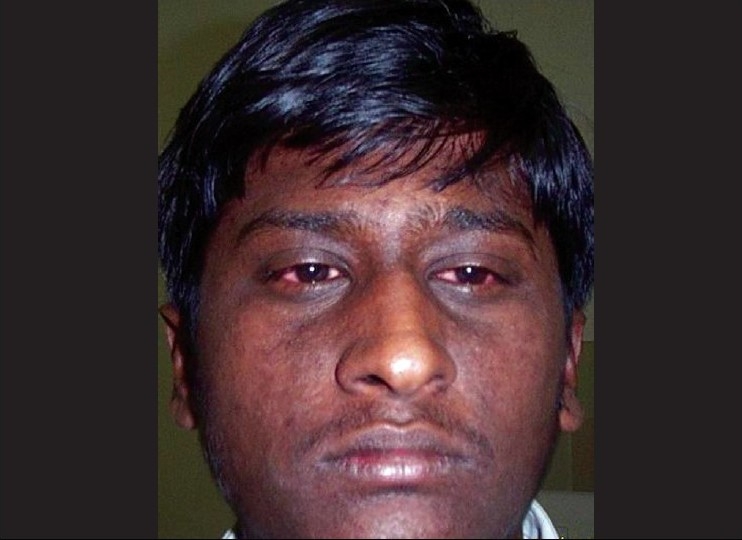
Photograph of the face showing papular skin eruptions

**Figure 2:**
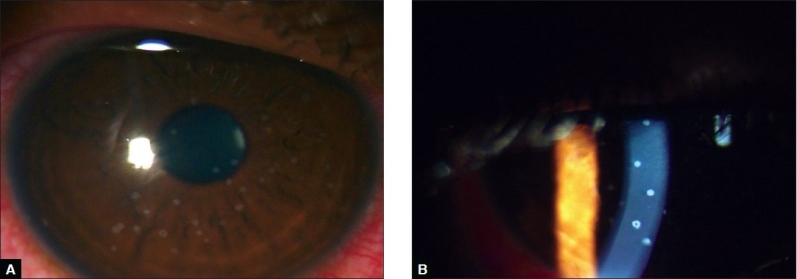
Slit-lamp photograph of the cornea in diffuse illumination (A) and slit illumination (B) showing multiple coin-shaped lesions and some lesions showing central clearing (X 1.5)

**Figure 3:**
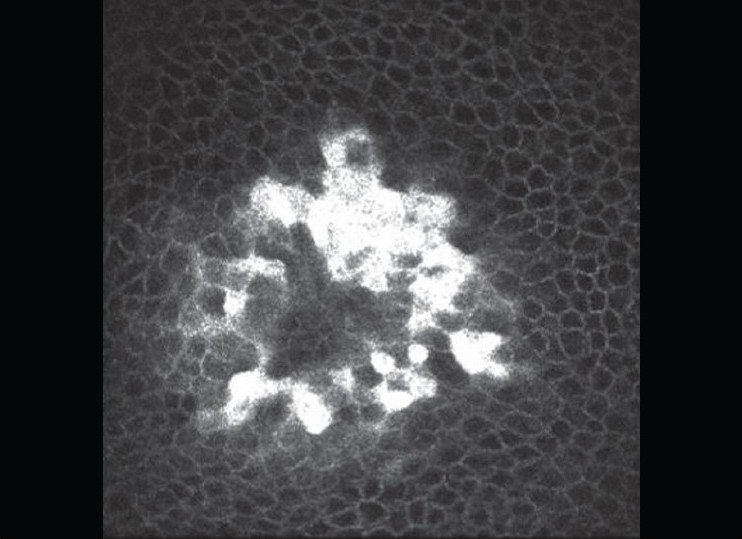
Confocal microscopy showing conglomerates of hyperreflective epithelial cells corresponding to the coin-shaped epithelial lesions (x 600)

**Figure 4:**
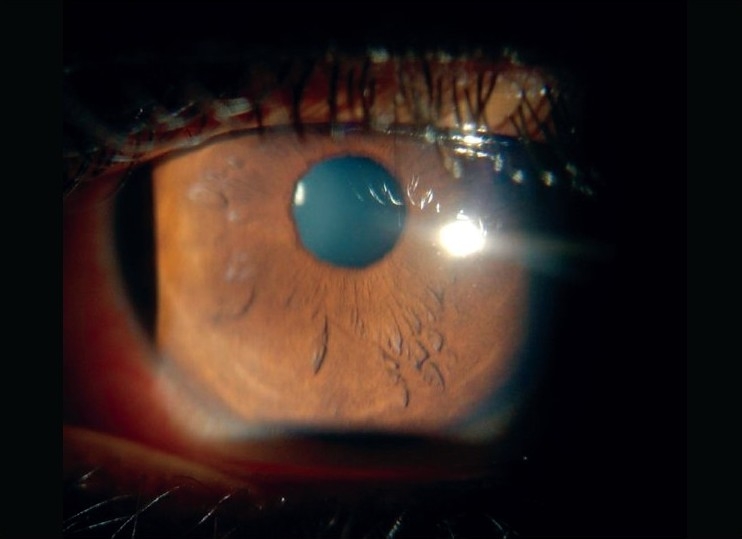
Slit-lamp photograph of the right eye in diffuse illumination showing disappearance of the corneal lesions following treatment (X 1)

On examination, he had papular skin eruptions on the face, arms and legs with two shallow painful ulcers in the buccal mucosa [Fig. 1]. His best-corrected visual acuity was 20/20 in both eyes. Slit-lamp examination in both eyes revealed multiple coin-shaped epithelial lesions, some showing central clearing [Fig. 2A, B]. There was no involvement of the stroma. Corneal sensation (tested with wisp of cotton) was normal. Rest of the anterior segment, intraocular pressures and fundus examination were normal. Confocal microscopy (HRT II, Rostock corneal module, Heidelberg, Germany) showed conglomerates of hyper-reflective epithelial cells corresponding to the coin-shaped epithelial lesions [Fig. 3]. The subepithelial nerve plexus, the stroma and endothelium were normal. He was started on oral prednisone, 60 mg/day slowly tapered by 10 mg/day over six weeks along with ocular lubricants (1% carboxymethyl cellulose eye drops, four times a day). The skin and corneal lesions [Fig. 4] resolved over six weeks. Repeat confocal microscopy showed normal epithelial cells, subepithelial nerve plexus, stroma with keratocytes and endothelium.

## Discussion

Erythema multiforme is an acute mucocutaneous hypersensitivity reaction characterized by a skin eruption, with or without oral or other mucous membrane lesions. EM has been classified into a number of different variants based on the degree of mucosal involvement and the nature and distribution of the skin lesions. EM major (toxic epidermal necrolysis) is more severe, typically involving two or more mucous membranes with more variable skin involvement – which is used to distinguish it from SJS, where there is extensive skin involvement and significant mortality and morbidity of 5-15%.[3] These features are considered to be sequelae of a cytotoxic immunologic attack on keratinocytes expressing non-self antigens. These antigens are primarily microbial (viral or bacterial infection) or drugs.[3] Cytotoxic effector cells, CD8^+^ T lymphocytes in the epidermis, induce apoptosis of scattered keratinocytes and lead to satellite cell necrosis.

On the other hand, EM minor typically affects no more than one mucosa, is the most common form and may be associated with symmetrical target lesions on the extremities. Herpes simplex virus-induced EM minor constitutes 15-60% of EM minor. The treatment is a combination of acyclovir and oral steroids.[3]

Ocular involvement in EM minor is rare. Unlike SJS, ocular findings in EM minor have not been very well-described in literature (Medline search).[1,2,4,5] We report an interesting ocular finding of coin-shaped lesions with some lesions showing central clearing confined to the epithelium of the cornea with no involvement of the underlying nerves or the stroma. Confocal microscopy findings show conglomeration of hyper-reflective lesions in the epithelium. The disappearance of these lesions following treatment is also documented on confocal microscopy. A probable immunological reaction targeted against the epithelial cells in the epithelium is speculated.

## Conclusion

In this case report, an interesting ocular finding of coin-shaped epithelial lesions with central clearing along with the confocal microscopy findings is reported in a patient following an acute attack of EM minor.
